# Translation and cross-cultural validation of the Turkish, Moroccan Arabic and Moroccan Berber versions of the 48-item Symptom Questionnaire (SQ-48)

**DOI:** 10.1192/j.eurpsy.2023.752

**Published:** 2023-07-19

**Authors:** V. Kovacs, I. Carlier, F. Zitman, A. van Hemert, E. Giltay

**Affiliations:** Psychiatry, Leiden University Medical Centre (LUMC), Leiden, Netherlands

## Abstract

**Introduction:**

First generation immigrants in many European countries have insufficient mastery of the host language to complete self-report questionnaires. To address this problem, we translated and validated Turkish, Moroccan Arabic and Moroccan Berber versions of the Dutch 48-item Symptom Questionnaire (SQ-48), which is a validated and clinical useful measure of psychopathology.

**Objectives:**

Therefore, this study describes the translation and cross cultural validation of the Turkish, Moroccan Arabic, and Moroccan Berber versions of the 48-item Symptom Questionnaire.

**Methods:**

Four samples were used: 1) psychiatric outpatients with Turkish or Moroccan background (n=150); 2) non-psychiatric subjects with Turkish or Moroccan background (n=103); 3) native Dutch psychiatric outpatients (n=189); 4) native Dutch non-psychiatric subjects (n=463). Data were analysed by confirmatory factor analysis and receiver operating characteristic curves.

**Results:**

The 253 psychiatric non-native patients and controls were on average 38,3 years old (SD 12,4), and 61% were women. Internal consistency of SQ-48 subscales across groups was adequate to high, the seven-factor structure of SQ-48 fitted the data adequately in the total sample and in each separate group, and AUC values showed acceptable to excellent discrimination. However, the mean severity scores for all SQ-48 subscales were significantly higher in the immigrant groups than those of the Dutch native group. We found full configural, metric and partial scalar invariance.

**Conclusions:**

Psychopathology measured by SQ-48 can largely be interpreted in the same way for persons from different immigrant backgrounds. However, cut-off values for Dutch natives should be ascertained using larger samples as these are likely higher than in Dutch psychiatric patients.

**Disclosure of Interest:**

None Declared

